# From Continental Priorities to Local Conservation: A Multi-Level Analysis for African Tortoises

**DOI:** 10.1371/journal.pone.0077093

**Published:** 2013-10-08

**Authors:** Pierluigi Bombi, Manuela D’Amen, Luca Luiselli

**Affiliations:** 1 Institute of Agro-environmental and Forest Biology, National Research Council, Monterotondo, Italy; 2 Centro Nazionale Biodiversità Forestale ‘Bosco Fontana’, Corpo Forestale dello Stato, Verona, Italy; 3 Centre of Environmental Studies Demetra s.r.l., Rome, Italy; 4 Eni s.p.a. Environmental Department, Rome, Italy; University of Western Ontario, Canada

## Abstract

Terrestrial tortoises are the most endangered group of vertebrates but they are still largely ignored for defining global conservation priorities. In this paper, we explored within a hierarchical framework the potential contribution of prioritization studies at the continental scale to the planning of local initiatives for the conservation of African tortoises at the regional level. First, we modeled the distribution of all the African tortoise species, we calculated three indicators of conservation priority (i.e. species richness, conservation value, and complementarity), and we carried out a gap analysis at continental scale. Second, we focused on the most important region for tortoise conservation and performed the same analyses at higher resolution. Finally, we compared the results from the two scales for understanding the degree to which they are complementary. Southern Africa emerged from the continental analysis as the most important region for tortoises. Within this area, the high-resolution analysis pointed out specific core sites for conservation. The relative degree of species protection was assessed similarly at the two different resolutions. Two species appeared particularly vulnerable at both scales. Priority indices calculated at high resolution were correlated to the values calculated for the corresponding cells at low resolution but the congruence was stronger for species richness. Our results suggest to integrate the calculation of conservation value and complementarity into a hierarchical framework driven by species richness. The advantages of large scale planning include its broad perspective on complementarity and the capability to identify regions with greatest conservation potential. In this light, continental analyses allow targeting fine scale studies toward regions with maximum priority. The regional analyses at fine scale allow planning conservation measure at a resolution similar to that required for the practical implementation, reducing the uncertainty associated with low resolution studies.

## Introduction

The limited availability of resources for biodiversity conservation worldwide makes essential targeting the practical efforts for maximizing the ratio between amount of biodiversity protected and money invested [1]. The process of systematic conservation planning [2] provides an efficient mechanism for identifying priorities, in terms of species and areas that should be the primarily focus of conservation initiatives. Several criteria have been adopted for defining such priorities [3,4] and most of them involve the analysis of species distributions. Like all spatially explicit processes, systematic conservation planning produces outcomes that depend on selected resolution and extent of analysis [5]. In addition, there is often a gap between the grain size used in conservation planning and the resolution at which conservation initiatives should be practically implemented. The selection of grain size in conservation planning is often an arbitrary choice and generally depends on the availability of high resolution data, the aim of the study, and/or computational constrains. On the other hand, the resolution at which conservation initiatives should be practically implemented is generally related to the scale of biological processes and to the extent of operative units of management [5,6]. For this reason, it is important to fully understand how the various attributes of biodiversity may change across spatial scales [7] for implementing conservation plans at different levels.

The resolution and the extent of analysis may directly influence the selection of conservation priorities throughout several phases. The grain size of species distribution maps introduces some uncertainty in the representation of the true distribution of species on the ground [8]. Such uncertainty may change with the method used for mapping species distributions [9,10]. Even if species maps are generated by distribution modeling techniques, the resolution still influences model performance [11,12]. The definition of specific conservation targets and the quantification of species protection levels are sensitive to over- or under-estimations of species distributions [10]. Since model errors propagate through the process of overlaying distribution maps [13], biodiversity patterns change with the grain size [14,15]. In addition, the size of spatial units, when it is different from the management units, influences the estimated representation of species in protected areas [16], introducing further uncertainty in the prioritization process. For these reasons, it is advised that the implementation of conservation efforts is conducted within a multi-scale framework for greater effectiveness.

A number of studies investigated conservation priorities at global and continental scale [4,17]. In Africa, these studies encompassed different taxa and utilized several approaches [18,19]. Most of the studies analyzed the distribution patterns of mammals, amphibians, birds, and snakes [18–20]. Only few studies investigated the conservation requirements of chelonians, focusing exclusively on freshwater species [10,21], with only a single study identifying priorities through a spatially explicit approach [10].

Many species of terrestrial chelonians live in Africa and South Africa has been identified as the global hotspot for tortoise diversity [22,23], housing almost one third of all the existing species in the world. Five genera and 14 species of Testudinids occur in the subcontinent [24] and three genera and 11 species of these are endemic to the area. Nevertheless, this group is still largely ignored in spatially explicit prioritizations. Since chelonians are the most endangered group of vertebrates in the world in terms of proportion of threatened species according to the International Union for Conservation of Nature (IUCN) Red List [22], improving conservation instruments for this taxon is of extreme importance, especially in Africa where pristine environments are collapsing and vanishing at a fast rate.

In this study we investigate the potential contribution of prioritization exercises at the continental scale to the definition of local priorities for the conservation of African tortoises at the regional level. To do this, we adopt a double-level approach. First, we evaluate the conservation priorities for tortoises in the whole of Africa and identify the highest priority region. Second, we focus on this high priority region to define core areas for tortoise conservation at an increased spatial resolution. For each species, we estimate the degree of protection at both scales and we analyzed the congruence in outcomes of continental and regional prioritizations. Our results must not be interpreted as ready-to-use indications for the practical implementation of conservation measures, but as a contribution to the understanding of how conservation planning may change across spatial scales. Our approach clarifies how different levels of analysis can interact, integrating the respective roles and providing a reciprocal complement.

## Methods

### Species locality records

We utilized species data provided by the “EMYSystem Global Turtle Database” [25]. These data consist of locality records from Iverson [26,27], collected in a web site by the “Terra Cognita” laboratory (Geosciences Department of Oregon State University in Corvallis, Oregon). Iverson’s works [26,27] are recognized as the state of the art and a milestone in the study of turtle distributions and are still used as references in fundamental papers in this field [24,28,29], being considered the most authoritative, comprehensive, and precise source of turtle distribution data, especially in Africa where frequent updating are not available. The EMYSystem Global Turtle Database contains distribution records for every species of freshwater and terrestrial chelonian that has been collected by a museum, private individual, or referenced in a publication. In the database, locations for sea turtles and for non-marine species described later than 1992 are not included. Locality records are mapped at a spatial resolution of 0.01 geographic degrees (about 1.1 km).

In total, we considered 16 species of Testudinidae: *Centrochelys sulcata*, *Chersina angulata*, *Homopus areolatus*, *Homopus boulengeri*, *Homopus femoralis*, *Homopus signatus*, *Homopus solus*, *Kinixys belliana*, *Kinixys erosa*, *Kinixys homeana*, *Kinixys natalensis*, *Malacochersus tornieri*, *Psammobates geometricus*, *Psammobates oculifer*, *Psammobates tentorius*, *Stigmochelys pardalis*. These species are only a very small fraction of the overall African biodiversity. Nevertheless, according to the Turtle Taxonomy Working Group of the IUCN/SSC Tortoise and Freshwater Turtle Specialist Group [24], this set of species represents almost the entire fauna of tortoises in Sub-Saharan Africa, only two species being excluded (*Kinixys lobatsiana*, *Kinixys spekii*). However, other studies consider additional taxa which were not recognized by Iverson et al. [25] and Rhodin et al. [24]. For instance, some authors considered *Kinixys nogueyi* as a valid West African species [30,31] rather than a subspecies of *Kinixys belliana* as in Rhodin et al. [24]. The number of usable records was extremely variable between species (mean = 81.50, SD = 96.62): there was only one record for *H. solus* and, for the other species, the range was from 11 records (for *K. natalensis*) to 316 records (for *K. belliana*).

### Environmental predictors

For both continental and regional analyses, we fitted species distribution models (SDMs) utilizing four groups of environmental predictors with high resolution (i.e. < 30 arc seconds): (i) 19 climatic variables from the WorldClim databank [32], (ii) two land morphology descriptors from the UNEP Environmental Data Explorer, (iii) three variables relating to water bodies from the FAO GeoNetwork, and (iv) one land cover variable from the JRC databank (see Table S1 for data sources and description). All the variables were re-sampled in ArcGIS 9.3 at the resolutions of one geographic degree for the entire continent and of five arc minutes for the regional analysis, by calculating the most common class for land cover and the average value for the other parameters. We calculated the variance inflation factor (VIF) to eliminate correlated variables [33]. VIF measures the degree of inflation of the unexplained variance due to the inter-correlation among independent variables [34,35]. We removed variables until all VIFs were below 5 [33], consequently we trained the models utilizing a subset of 11 and 10 predictors for continental and regional analyses respectively (Table S1). This process reduced the comparability of distribution models between scales but, at the same time, allowed to optimize the models by avoiding the introduction of biases due to correlation between predictors.

### Modeling procedure

Because the choice of algorithm influences the performance of SDMs, we adopted an ensemble forecasting approach [36]. We used five different techniques for modeling habitat suitability in the R-based (version 2.8.1 [37]) package BIOMOD [38], at two spatial resolutions for the continental and the regional analyses (one degree and five arcmin respectively). We fitted Generalized Boosting Model (GBM [39]), Generalized Linear Models (GLM [40]), Multiple Adaptive Regression Splines (MARS [41]), Flexible Discriminant Analysis (FDA [42]), and random forest for classification and regression (RF [43]) on presence and pseudo-absence data for all the species occurring in the considered area. Generalized Boosting Model are models that combine two different techniques – regression trees and boosting – to optimize predictive performance [39], Generalized Linear Models are regression models that allow non-linear distributions for the response variable [40]. Multiple Adaptive Regression Splines are models that combine linear regression, mathematical construction of splines, and binary recursive partitioning to produce a local model [41]. Flexible Discriminant Analysis are classification models based on the well-known linear discriminant analysis [42]. Random Forest for classification and regression are models that create a suite of models using a classification and regression tree [43]. We selected 500 (in the continental analysis) and 200 (in the regional analysis) random cells across the study area as pseudo-absences in order to have a good representation of the background conditions. With a fixed number of pseudo-absence points, the prevalence changed across species and was proportional to the number of species presences. We validated each model by calculating the Area Under Curve (AUC) through a 10-fold cross-validation procedure [44]. For each species, we produced a single consensus model by calculating the weighted average of any single model with AUC > 0.7 [36] using AUC values as model weights.

We converted to predicted presence/absence the continuous values of habitat suitability (HS) predicted by the consensus models according to the minimal predicted area criterion [45,46], i.e. HS threshold selected to achieve a sensitivity (i.e. the true positive fraction) of 0.9. We chose this threshold, by producing a set of thresholds from different criteria (i.e. maximum percentage of presence and absence correctly predicted, maximum kappa, maximum TSS) and selecting the criterion that produced the highest threshold, in order to reduce the rate of commission error in SDMs. Grid cells where a species was recorded were treated as presences regardless of the model predictions. In order to exclude the areas well beyond the known range of a species, we removed all areas that do not currently contain locality records and are isolated from other areas that do contain records by a barrier of unsuitable habitat wider than the mean distance between closest pairs of locality records for the species. This procedure produced one continuous area or few separated areas per species that can be considered as the best approximation of the true species distribution at the given resolution. In the case of *Homopus solus*, which has only one record in our dataset, we considered the unique occupied cell as the entire distribution at both the resolutions.

### Measures of conservation priority

We performed all analyses of conservation priority at both continental and regional scale. We used species distributions to estimate three indicators of conservation priority for each grid cell in the study areas: (i) species richness, (ii) conservation value, and (iii) complementarity with existing reserves. Species richness was derived by simply counting the number of species estimated to occur in each cell. In order to evaluate conservation value and complementarity of the grid cells we used an approach based on the principle of irreplaceability [47]. We used the C-Plan Systematic Conservation Planning System, Version 4 to predict irreplaceability [48]. Irreplaceability of each cell was estimated as the number of possible combinations of cells that include the focal cell and meet a predefined set of specific conservation targets, but which would not meet the targets if the focal cell was removed, divided by the total number of possible combinations that meet the targets (see 3 for details). Values close to 1 indicate sites difficult to replace, often containing species endemic to those sites, while values close to 0 indicate easily replaceable sites, containing only widely distributed species. Following the suggestion by Pressey et al. [47], in order to estimate the ‘landscape’ of conservation value, we calculated the irreplaceability on the entire study areas, considering all the cells as non-protected. In order to estimate the potential contribution of each available cell to the improvement of the established network of protected areas, we calculated the level of complementarity of the cells as the irreplaceability value calculated for unprotected cells by taking into account the existing reserves [47].

Dealing with large scale datasets, a problem arises when reserve boundaries, which are mapped as polygons, should be matched with species distribution maps in regular grids [16]. In these cases, it is necessary to define a threshold percentage of intersection between cells of distribution maps and polygons of reserves boundaries for determining whether a grid cell should be considered protected or not. This choice is crucial, because it potentially generates an over- or under-estimation of the protection level. For solving this issue, we tested different thresholds, using the 2010 WDPA annual release of reserve boundaries [49]. Protected areas with only a point location were mapped as circles with appropriate surface. At both the scales of analysis, we chose the thresholds that selected a number of cells with a total surface equal to the total surface of reserves in the study areas [50]. On the basis of this approach, we considered protected any cell with a proportion of park coverage larger than 36.95%.

In the gap analysis, we determined whether each species met a conservation target set in terms of percentage of its distribution intersecting the current reserve system. We defined the conservation target for each species as the minimum number of cells that should be protected to consider the species sufficiently represented. We established the conservation targets for species on the basis of range sizes, following Rodrigues et al. [17]. We set the conservation target to 100% of the cells protected for the species with the smallest extent of occurrence. On the other hand, we set to 5% the conservation target of the most widespread species. Targets for all species with intermediate range sizes were calculated by interpolating the extreme range size targets using a linear regression on the log-transformed number of occupied cells. We considered those species not represented in any protected area as total gap species and species that met only a portion of their conservation target as partial gap species [17].

### Cross-levels comparison

In order to explore the potential cross-scale interaction, we compared the outcomes of continental and regional prioritizations. More specifically, we evaluated if (i) the relative degree of species protection and (ii) the land priority values were congruent at the two resolutions. To do this, we adopted a null models approach, contrasting the observed correlation coefficient *r* with those simulated by 3 × 10^4^ random Monte Carlo permutations in EcoSim 7.0 [51]. This number of permutations ensures that algorithm biases are avoided [52]. The index (r) was calculated for the original data as well as for the simulated matrices and results were compared, calculating the probability (*P*) of the null hypothesis that the observed index (*r*_*obs*_) was drawn at random from the distribution of the simulated indices (*r*_*exp*_) [53]. This means that the observed correlation in the data does not reflect real patterns, but represents chance variation or sampling effects. Non-random correlations were assumed when *P*_*Fobs≥Fexp*_ ≤ 0.05 [54]. In addition, we defined as priority sites the envelope of the cells with priority score higher than the threshold that selects a number of cells as close as possible to the best 1%, and we measured if the priority sites identified at high resolution were nested across the priority sites identified at low resolution.

## Results

### Continental analysis

At the continental scale, we obtained high validation scores for all the species (mean AUC ± SD = 0.894 ± 0.041). One half of the species appeared localized to relatively small areas (< 100 cells) and most of them are endemic to Southern Africa (Table 1). On the contrary, we evidenced only three very widespread species (> 500 cells). The protection provided to tortoises by the African network of protected areas was highly variable (Table 1). Similarly, the percentage of target met by each species changed. Three species met their respective conservation targets but, at the same time, two species were not represented at all in the reserve system (Table 1).

**Table 1:** Species range extent, percentage of range protected, conservation target, and percentage of target met for low and high resolutions.

**Species**	**Low resolution**					**High resolution**			
	**Range**	**% range protected**	**Target**	**% target met**		**Range**	**% range protected**	**Target**	**% target met**
*Centrochelys sulcata*	644	15.68	77	-					
Chersina angulata*	56	19.64	25	*43.52*		4168	18.09	680	-
Homopus areolatus*	66	3.03	28	*7.06*		3401	9.38	624	51.12
Homopus boulengeri*	137	12.41	45	*37.71*		4129	6.01	677	*36.62*
Homopus femoralis*	77	3.90	31	*9.56*		3620	8.51	642	*48.01*
Homopus signatus*	24	0.00	14	***0.00***		1319	8.87	367	*31.84*
Homopus solus*	1	0.00	1	***0.00***		1	0.00	1	***0.00***
*Kinixys belliana*	1082	18.02	54	-		2782	25.27	567	-
*Kinixys erosa*	343	14.58	70	71.59					
*Kinixys homeana*	85	15.29	34	*38.73*					
Kinixys natalensis*	44	4.55	21	*9.36*		704	10.94	241	*32.01*
*Malacochersus tornieri*	212	22.64	57	84.02					
Psammobates geometricus*	5	40.00	4	51.25		263	18.25	116	*41.43*
Psammobates oculifer*	156	17.31	49	55.23		12189	16.17	674	-
Psammobates tentorius*	122	7.38	42	*21.40*		7469	10.24	781	98.01
*Stigmochelys pardalis*	661	23.60	77	-		12852	16.67	643	-

Percentages of target met larger than 100% are represented by a dash. Values lower than 50% are presented in italics and percentages equal to 0 are highlighted in bold italic. Starred species are endemic to Southern Africa.

**Figure 1:**
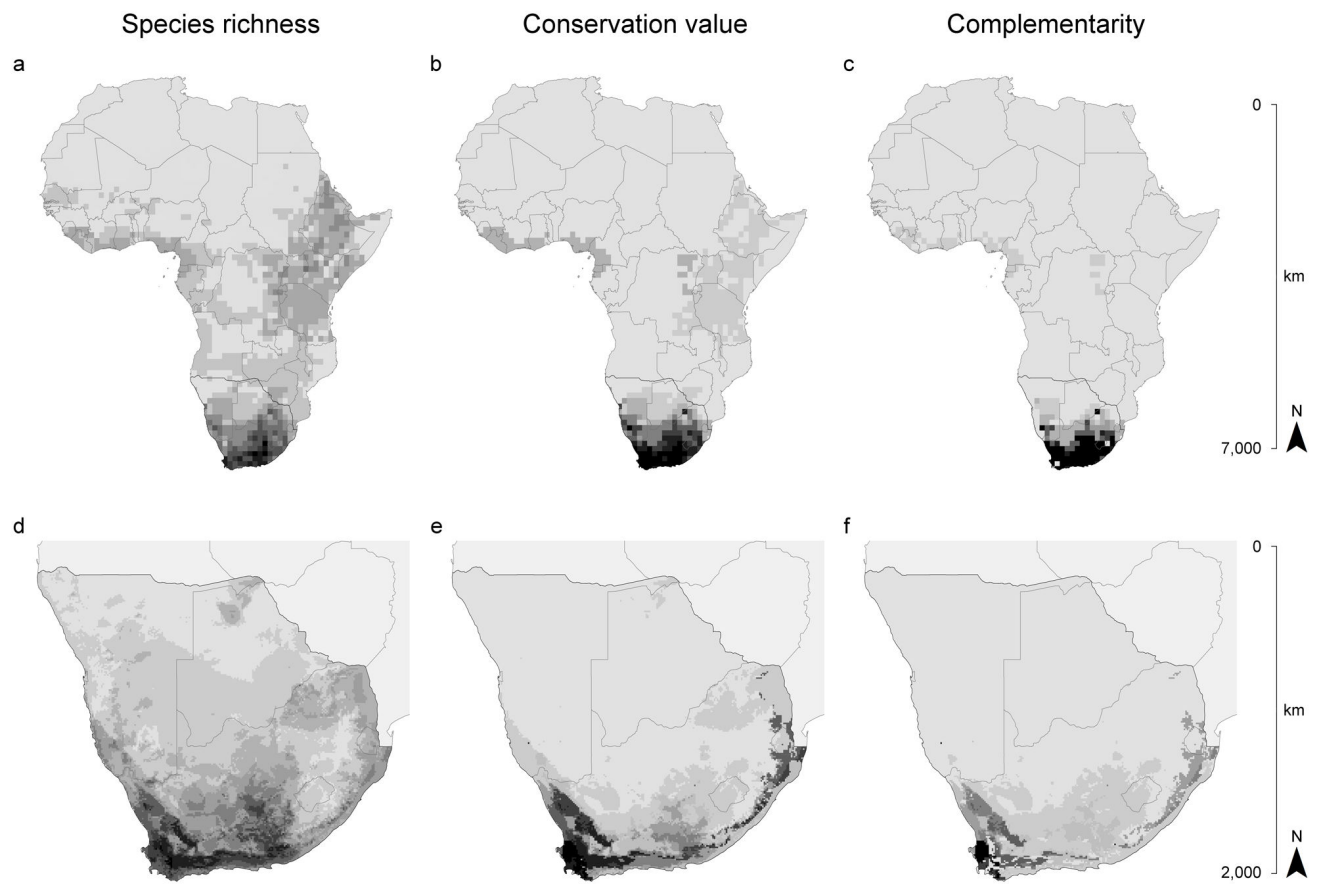
Continental and local conservation priorities. Geographic patterns of: total species richness (a,d), conservation value (i.e. irreplaceability calculated considering all the cells as non-protected; b,e), and complementarity (i.e. irreplaceability calculated considering the presence of the existing reserves; c,f) calculated at low resolution for the entire Africa (a,b,c) and at high resolution for Southern Africa only (d,e,f). Darkness of the pixels is proportional to cell values (see text for ranges).

The combination of species models produced very clear patterns of species richness, conservation value, and complementarity. We observed the highest values of species richness in Southern Africa (especially in central and southern South Africa) (see Figure S1 for place names), where all but one the 3.6% most specious cells (≥ 5 species; max: 9 species) were restricted (Figure 1a). Secondary peaks of tortoise diversity were also present in East Africa (from Eritrea to northern Tanzania) and along the Gulf of Guinea (from Sierra Leone to Ghana and from Nigeria to Gabon) (Figure 1a). Similarly, all but one the best 6.7% cells in terms of conservation value were located in Southern Africa (especially in central and southern South Africa and southern Namibia) (Figure 1b). At the same time, East Africa reduced its relative importance and the Gulf of Guinea maintained its secondary prominence if conservation value was considered (Figure 1b). When we considered the existence of reserves for calculating the complementarity value, Southern Africa consolidated its great importance, containing the best 6.1% cells (Figure 1c). On the contrary, the Gulf of Guinea and, especially, East Africa further reduced their relative importance (Figure 1c).

### Regional analysis

Southern Africa emerged from the continental analysis as the most important region for tortoises. High resolution models for this region obtained high validation scores (mean AUC ± SD = 0.879 ± 0.023). Ten of the 12 species that occur in Southern Africa are endemic to this region and we evidenced that 40% of these species were restricted to a small area (< 2000 cells) (Table 1). At the same time, two species were very widespread across the region (> 10000 cells) (Table 1). At the regional scale, the protection provided by the reserve network was similar to the level of the entire Africa but slightly more constant (Table 1). Similarly, we estimated a great variation among the percentages of target met by each species. Only one species was not represented in any protected area and four other species met their respective conservation targets.

When the priority level was quantified at high resolution across Southern Africa, specific areas emerged as core sites for tortoise conservation. The 4.1% most specious cells (> 5 species; max: 8 species) corresponded to the Cape Fold Mountains, the south-western ranges of the Great Escarpment, and the middle and upper basin of the Great Fish River (Figure 1d) (see Figure S1 for place names). Some other areas along the coasts and the eastern border of South Africa, in south-western Namibia, and in northern Botswana appeared as secondary peaks of species richness. In terms of conservation value, the highest levels (best 0.6% cells) were evidenced in the coastal area south-west of the Cape Fold Mountains and in a single cell in the south-western Namibia (Figure 1e). The Cape Fold Mountains themselves and the south-western ranges of the Great Escarpment maintained an outstanding value (best 4.0% cells), which was also obtained by the south-eastern slopes of the Drakensberg mountain range. On the contrary, the basin of the Great Fish River reduced its importance. Measuring the value of complementarity with respect to the existing reserve network, the coastal area south-west of the Cape Fold Mountains and the cell in south-western Namibia further increased their importance with respect to the other zones (Figure 1f), containing the best 0.4% cells.

### Cross-levels comparison

The relative degree of species protection was assessed similarly at the two different resolutions. For the species endemic to Southern Africa, the percentages of protected range measured at low and high resolutions were strongly correlated (arc-sine transformed % protected range_(LR vs HR)_: *r*_*obs*_ = 0.699, *P*_*obs ≥ exp*_ = 0.006). Similarly, considering that species with the percentage of protected range ≥ 100% meet their targets and have, consequently, the percentages of target met = 100%, the percentages of target met calculated at high and low resolutions were correlated (arc-sine transformed % target met_(LR vs HR)_: *r*_*obs*_ = 0.666, *P*_*obs ≥ exp*_ = 0.008). The relative priority values of the cells at high resolution were similar to the relative priority values of the corresponding cells at low resolution. All the three indices of priority (species richness, conservation value, and complementarity) calculated at high resolution were correlated to the values calculated for the corresponding cells at low resolution (*r*_*obs*_ = 0.709, *r*_*obs*_ = 0.336, and *r*_*obs*_ = 0.374 respectively, *P*_*obs ≥ exp*_ ≤ 0.001 in every case). The highest priorities identified at the regional level were nested across the priorities defined at the continental scale. Almost all (98.7%) the 1.5% most specious cells at high resolution were contained in the 2.3% most specious cells at low resolution. At the same time, 94.3% of the 0.9% most valuable cells and 90.7% of the 1.9% most complementary cells at high resolution were contained in the best 0.9% and 1.9% cells at low resolution in terms of conservation value and complementarity respectively.

## Discussion

Although both spatial resolution and extent of analysis in prioritization exercises depend on the level of detail desired, their choice is generally constrained by the availability of data and resources. Coarse data sets are usually available at continental scale, but this information alone is of little use for practical implementations. On the contrary, fine-scale presence datasets are usually limited to small areas. In addition, even if detailed records would be available, identifying conservation priorities at high resolution on very large surfaces (e.g. continents) can require an overwhelming mass of calculations, which are intractable with standard hardware instruments. The constraints on fine-scale mapping across large regions can be overcome by a hierarchical approach, if one can show the capability of large scale planning to identify regions with greatest conservation potential [14]. Here, we observed congruence in the geographic pattern of priorities and species conservation needs identified at the continental and regional levels. This congruence has been already observed previously [14,55], further supporting the use of this approach.

In our study, the correlation observed between regional and continental values is stronger for species richness than for the other indices. Similarly, the degree to which the most important sites at the regional level are nested in the most important sites at the continental level is higher for species richness than for the other indices. Even the visual inspection of the priority maps reveals that the patterns of species richness in Southern Africa determined by the two levels of analysis coincide more strictly than the patterns of conservation value and complementarity. This dissimilarity is due to the sensitivity of the irreplaceability calculation to different sources of uncertainty (e.g. definition of protected units, community composition, extent of study area). Operatively, the scale-dependence of conservation value and complementarity suggests to use the geographic pattern of species richness for linking the different levels of analysis in a hierarchical framework of prioritization.

The two levels of analysis quantified similarly the degree of protection for each species. Most of the species are under-represented in the protected areas with respect to our conservation targets in the entire continent and in Southern Africa as well. Two species appear particularly neglected at both scales of analysis. Although this evidence may be due to the mapping method used for *Homopus solus*, which can underestimate its real distribution, this species does not occur in any reserve and is listed as Vulnerable in the IUCN Red-List [56]. Similarly, *Homopus signatus* is completely uncovered at low resolution and meets only one third of its target at high resolution. However, this species is listed as ‘Lower Risk/Near Threatened’ in the Red-List [56]. These two species should be considered as priorities in future management actions and deserve further studies for clarifying their conservation status.

The percentages of target met by species were slightly higher at local than at continental scale. If not a byproduct of chance, this small difference may be due to the criterion adopted for setting the conservation targets, which selected less demanding targets at high resolution. However, these values as well as the percentages of range protected estimated at high resolution are proportional to the corresponding values at low resolution. In addition, the percentages of range protected are also similar in value. These factors suggest that the criterion for conservation targets should be changed with the study resolution but also that our approach for defining protected cells is robust to cross-scale variations. Therefore, continental analyses can represent a preliminary instrument for defining priority species at regional level.

South Africa emerged as by far the most important area for tortoise conservation at the continental level. This finding does agree with the outcomes of other studies [22,23]. The most important areas for tortoises identified in Southern Africa correspond to global priorities for ecoregion conservation. The Cape Fold Mountains and the coastal area south-west of them belong to one of the Global 200 ecoregions (i.e. ‘Fynbos’ [57]) and to one of the global biodiversity hotspot (i.e. ‘Cape floristic province’ [4]). Similarly, the south-western ranges of the Great Escarpment match with the ‘Namib and Karoo deserts and shrublands’ ecoregion [57] and the ‘succulent karoo’ hotspot [4]. The importance of the coastal area south-west of Cape Fold Mountains in terms of both conservation value and complementarity is further increased by the presence of several small ranging species and the relative lack of protected areas. Future plans for the conservation of tortoises in Southern Africa should be focused especially on these areas, where detailed studies will be required in order to identify specific sites and practical guidelines for species management. These detailed studies will have to (i) identify species of conservation concern, (ii) define species distribution at very high resolution by talking to local experts, (iii) quantify the level of protection actually provided by the existing reserves to the target species, (iv) highlight demographic and life-history traits for these populations, and (v) work with stakeholders to identify sites where conservation action is feasible. Furthermore, this area is expected to require high costs for conservation implementation [58], reinforcing the importance of optimization strategies for tortoise protection and of large investments from funding agencies.

On the other hand, the importance of this region does not match with the conservation assessment for other animal groups in Africa [10,19]. A mismatch in the geographic localization of high priority areas for different animal groups has already been shown elsewhere [50,59]. The prioritization approach often aims to preserve all species from a selected area considering together different taxonomic groups. Treating in a unique step different taxa may underestimate the needs of single, less numerous and more specialist groups. Moreover, this could be true not only from a taxonomical point of view, but also it can be related to the ecological needs. Indeed, the priorities we identified for terrestrial tortoises are completely different from those for freshwater turtles [10]. The evidence that Southern Africa represents a specific priority for tortoises but not for other wildlife should encourage the conservation agencies in this area (e.g. national administrations, country-based and international organizations, managers of protected areas) to systematically focus their efforts on terrestrial chelonians.

Other regions are also relatively important for tortoise conservation at the continental level. The Gulf of Guinea and especially East Africa reduce their relative importance when conservation value is considered in place of species richness. This reduction is due to the large distribution of the species occurring in these areas that, therefore, give a small contribution to the irreplaceability of the occupied cell. This reduction could be smaller if different taxonomic arrangements are considered. Indeed, the splitting of the *Kinixys belliana* group would reduce the range extent of the different species. The further reduction of importance of East Africa, and secondarily of the Gulf of Guinea, in terms of complementarity with respect to the existing reserves testifies that the large protected areas established for protecting other wildlife in these regions obtained a sufficient representation also for tortoises. Both Gulf of Guinea and East Africa have been highlighted as hotspots for mammals, birds, amphibians, and other reptiles [10,18,19,60]. In this light, further studies at higher resolution are required in these areas for understanding whether tortoises should be considered in future conservation planning.

## Conclusions

Performing two levels of prioritization analyses can provide reciprocal complements. On the one hand, continental analyses allow us to overcome the ‘tyranny of the local’ (sensu [61]), which consists in overlooking important areas at the large scale when reserves are planned locally. On the other hand, regional analyses allow planning conservation initiatives at the same resolution as their practical implementation. This reduces the uncertainty due to modeling species distributions and defining priorities at low resolution. When only coarse-grain distribution data are available (e.g. atlas maps), downscaling techniques can be used for increasing the resolution of spatial predictions in local analyses [62]. Thus, organizing multiple levels of analysis into a hierarchical framework of prioritization can represent a helpful strategy for overcoming the limits of the single, independent levels and further improving the overall efficacy of conservation planning processes. 

## Supporting Information

Figure S1 **Geographic location of place names used in the text.**
(DOCX)

Table S1 **Variables selected for modelling species distribution at low and high resolution and relative data source (see main text for details).**
(DOCX)
