# Supplementary figures and images for: From Continental Priorities to Local Conservation: A Multi-Level Analysis for African Tortoises
Source: PLoS One. 2013 Oct 8;8(10):e77093. doi: 10.1371/journal.pone.0077093 (PMC3792937; doi:10.1371/journal.pone.0077093)

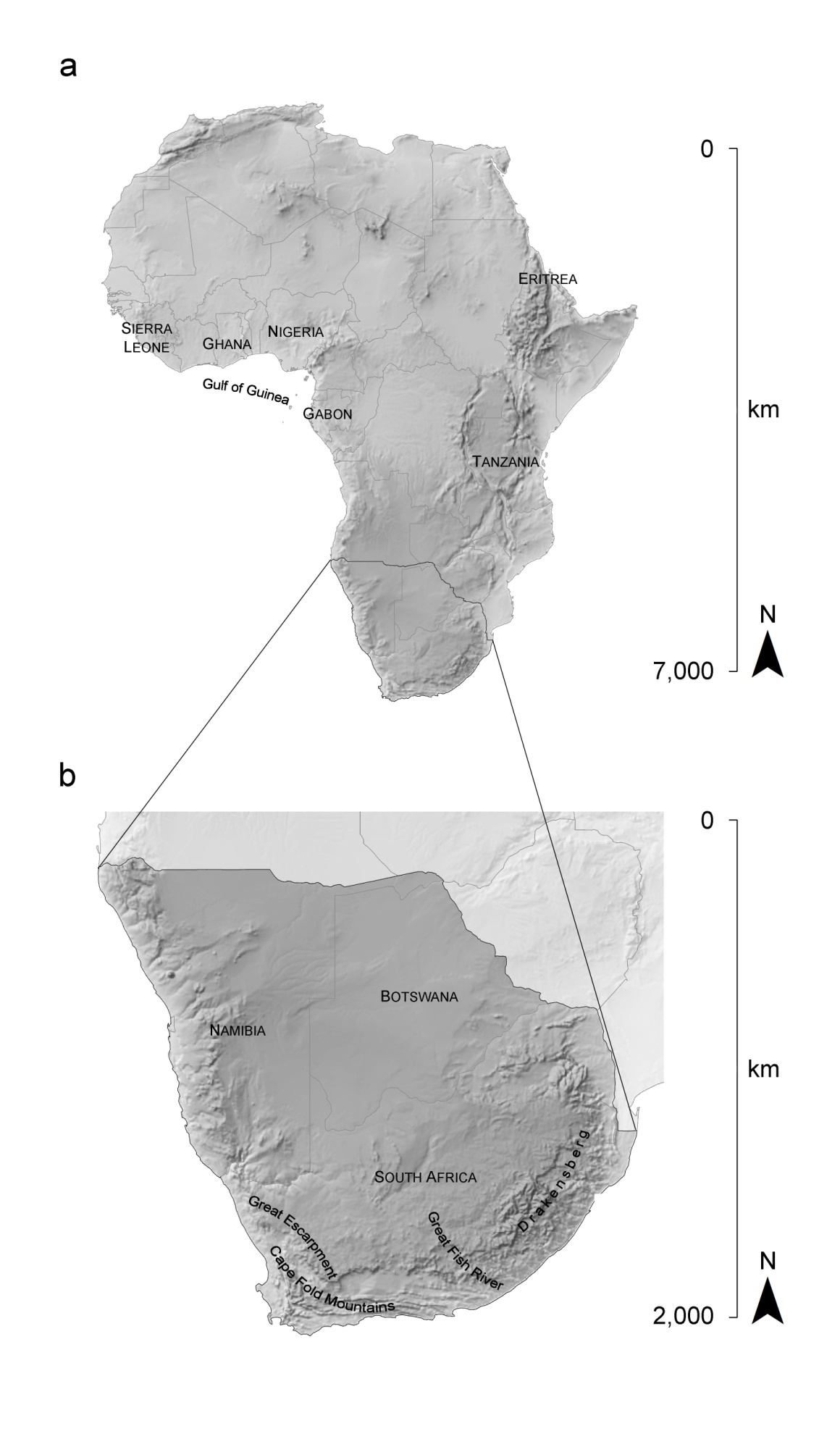


Figure S1. Geographic location of place names used in the text.

Supplement: Figure S1 — Geographic location of place names used in the text. (DOCX) [file pone.0077093.s001.docx]
