# Supplementary material for: From Continental Priorities to Local Conservation: A Multi-Level Analysis for African Tortoises
Source: PLoS One. 2013 Oct 8;8(10):e77093. doi: 10.1371/journal.pone.0077093 (PMC3792937; doi:10.1371/journal.pone.0077093)
Supplement: Table S1 — Variables selected for modelling species distribution at low and high resolution and relative data source (see main text for details). (DOCX) [file pone.0077093.s002.docx]

Table S1. Variables selected for modelling species distribution at low and high resolution and relative data source (see main text for details).

| Variable | Data Source | Low Res | High Res |
| --- | --- | --- | --- |
| Annual Mean Temperature | http://www.worldclim.org | X | X |
| Temperature Annual Range | http://www.worldclim.org |  | X |
| Mean Temperature of Wettest Quarter | http://www.worldclim.org | X |  |
| Mean Temperature of Driest Quarter | http://www.worldclim.org | X |  |
| Mean Temperature of Warmest Quarter | http://www.worldclim.org |  | X |
| Mean Temperature of Coldest Quarter | http://www.worldclim.org |  | X |
| Annual Precipitation | http://www.worldclim.org | X | X |
| Precipitation Seasonality (Coefficient of Variation) | http://www.worldclim.org |  | X |
| Precipitation of Wettest Quarter | http://www.worldclim.org | X |  |
| Precipitation of Driest Quarter | http://www.worldclim.org | X |  |
| Precipitation of Coldest Quarter | http://www.worldclim.org |  | X |
| Elevation | http://geodata.grid.unep.ch | X | X |
| Land Slope | http://geodata.grid.unep.ch | X |  |
| Linear Distance from the Closest Perennial River | http://www.fao.org/geonetwork | X | X |
| Hydrographical Basin | http://www.fao.org/geonetwork | X |  |
| Land Cover | http://bioval.jrc.ec.europa.eu/products/ glc2000/data_access.php | X | X |
